# Adhesive Strength in Dentin Conditioned with 18% Ethylenediaminetetraacetic Acid versus 35% Phosphoric Acid: In Vitro Study with 1-Year Artificial Aging

**DOI:** 10.3390/polym14204291

**Published:** 2022-10-12

**Authors:** Esther Alcántara-Obispo, Flor Santander-Rengifo, Marysela Ladera-Castañeda, Carlos López-Gurreonero, Antonieta Castro Pérez-Vargas, Alberto Cornejo-Pinto, Luis Cervantes-Ganoza, César Cayo-Rojas

**Affiliations:** 1 School of Stomatology, Universidad Privada San Juan Bautista, Lima 15066, Peru; 2 Academic Program of Dentistry, Universidad Peruana de Ciencias Aplicadas, Lima 15066, Peru; 3 Faculty of Dentistry and Postgraduate School, “Grupo de Investigación Salud y Bienestar Global”, Universidad Nacional Federico Villarreal, Lima 15001, Peru; 4 School of Stomatology, Universidad Científica del Sur, Lima 15067, Peru; 5 Faculty of Stomatology, Universidad Inca Garcilaso de la Vega, Lima 15084, Peru

**Keywords:** phosphoric acid, ethylenediaminetetraacetic acid, resin composite, bulk fill resin, adhesive strength, shearing

## Abstract

The success and longevity of a resin composite restoration is determined by its good bonding to the tooth structure, with the adhesion being a challenge to dentin due to its complexity and structural heterogeneity. The present study aimed to compare the adhesive strength of dentin conditioned with 18% ethylenediaminetetraacetic acid (EDTA) versus 35% phosphoric acid (H_3_PO_4_) in human premolars. Materials and Methods: This in vitro experimental study was performed on 40 human premolars. The occlusal thirds were sectioned and randomly placed into four groups according to the type of dentin conditioning: Group 1 (control), Group 2 (18% EDTA), Group 3 (35% H_3_PO_4_) and Group 4 (18% EDTA plus 35% H_3_PO_4_). Then, 10,000 thermocycles between 5 +/− 2 °C and 55 +/− 2 °C were applied. Adhesive strength was tested by shearing with a digital universal testing machine at a crosshead speed of 0.75 mm/min. The values obtained were analyzed in megapascals (MPa). The mean and standard deviation were used as measures of central tendency and dispersion. In addition, a one-factor intergroup ANOVA test was applied with Tukey’s post hoc test considering a significance level of *p* < 0.05. Results: The 18% EDTA and 18% EDTA plus 35% H_3_PO_4_ showed significantly higher adhesive strength compared to the control group that did not receive dentin conditioning (*p* = 0.047 and *p* < 0.001, respectively). However, the group conditioned with 18% EDTA did not present significant differences compared to the group conditioned with 35% H_3_PO_4_ (*p* = 0.997). In addition, the group conditioned with 18% EDTA plus 35% H_3_PO_4_ showed significantly higher adhesive strength compared to the groups conditioned with 18% EDTA (*p* = 0.002) and 35% H_3_PO_4_ (*p* = 0.001). Conclusion: The adhesion of bulk fill resin composite to dentin was favorable when preconditioning was performed using 18% EDTA followed by 35% H_3_PO_4_. In contrast, when both etchants were used separately, the bulk fill resin composite showed similar bond strength values in both cases, but significantly lower compared to their sequential application.

## 1. Introduction

The adhesive strength of resin composite restorations is a major concern for the dental professional as their failure is often due to a lack of adhesive strength in the hybrid layer at dentinal level [1,2,3,4]. Therefore, various types of cavity conditioners, such as 35% or 37% phosphoric acid (H_3_PO_4_), sodium hypochlorite, hydrogen peroxide or ethylenediaminetetraacetic acid (EDTA), among others [5,6,7,8,9,10,11,12], have been used to improve the adhesion of resin composites to the dentin substrate. These conditioners have been tested by in vitro shear [13] or microtension [1,5,6,14] with a universal testing machine to determine adhesive strength.

The 35% H_3_PO_4_ gel has been generally used as dental conditioning agent for cavity preparations because it removes dentin sludge and provides microretention on the dental substrate, giving the hybrid layer considerable adhesive strength. [5,15] However, it is important to mention that etching dentin with phosphoric acid decreases the calcium concentration because the extrafibrillar and intrafibrillar minerals dissolve, making the collagen fibers very susceptible to dehydration. [16,17,18]

EDTA is a mild chelating agent with almost neutral pH (pH = 7.4) [7,19,20,21] compared to phosphoric acid, which causes different effects on dentin depending on its concentration and exposure time. Its proven conditioning action causes less and more superficial dentin demineralization, chelating calcium ions while preserving and avoiding alterations of native fibrillar collagen, and therefore less alteration of dentin proteins, such as collagen fibers, that retain most of the intrafibrillar mineral content. This greater amount of residual apatite crystals in the collagen matrix improves its longevity [6,16,22,23] and also partially removes the smear layer up to 0.5 to 5 µm, keeping 30% of it inside the tubules without causing morphological alterations on the dentin surface. It also favors the opening of dentinal tubules for the formation of resin tags when placing the adhesive in the hybridization technique [7,12,15,21,24,25]. Cederlund et al. [26] reported that EDTA treatment increased shear bond strength, while Sauro et al. [14] reported that conditioning the smear layer with EDTA produced a less porous resin–dentin interface, resulting in a favorable effect on shear bonding. It should be noted that EDTA is an organic tetracarboxylic acid derived from ethane with the ability to chelate metal ions, with preference for Ca, Mg, Mo, Fe, Cu and Zn ions [5]. The interface created by this type of dentin conditioning presents lower degradation values because the greater number of crystals present in the collagenous matrix prevents its denaturation and promotes dentin remineralization [16]. A milder alteration of dentin proteins, compared to conditioning with phosphoric acid, allows the collagen to retain more apatite crystals, which could favor a greater mechanical microretention of the bonding agent when it is light cured. In addition, EDTA has an inhibitory effect on metalloproteinases (MMPs) that are bound to the demineralized dentin matrix, blocking their enzymatic action by chelating the ionic cofactors necessary for the catalytic activity of these enzymes and producing more stable adhesive interfaces [1,27].

The present study is important because, if it is demonstrated that 18% EDTA gel increases the adhesive strength of light-curing resin composites, it could be a good option to favor the permanence and longevity of dental restorations by preventing their detachment during masticatory action.

For the above reasons, the present study aimed to determine which is the best dentin conditioning agent for conferring greater adhesive strength to resin composite restorations. For this purpose, 18% EDTA gel versus 35% H_3_PO_4_ gel were compared, controlling the variables “type of adhesive” and “type of light-curing resin composite”. It was considered as null hypothesis that restorations with resin composite in dentin conditioned with 18% EDTA would not present significant differences in adhesive strength when compared to dentin conditioned with 35% H_3_PO_4_ in human premolars.

## 2. Materials and Methods

### 2.1. Type of Study and Delimitation

This experimental in vitro, cross-sectional and analytical study was carried out at the Stomatology School of the Universidad Privada San Juan Bautista and at the High Technology Laboratory Certificate (ISO/IEC Standard: 17025), in Lima, Peru from January to March 2022. This study considered the CRIS Guidelines (Checklist for Reporting In Vitro Studies) [28].

### 2.2. Sample Calculation and Selection

The total sample was 40 human premolar teeth equally distributed in four groups under simple random sampling without replacement (n = 10). The sample size was calculated using the data obtained in a pilot study prior to the final experiment with 5 sample units per group from a one-way analysis of variance formula in the statistical software G*Power version 3.1.9.7, obtaining an effect size (f) = 1.386, considering a significance level (α) = 0.05 and a statistical power (1-β) = 0.80.

Inclusion criteria are as follows:

- Human premolars extracted in the last three months prior to the study.
- Upper or lower human premolars extracted for orthodontic purposes.
- Human premolars without dental caries.
- Human premolars without previous fillings or sealants.

Exclusion criteria are as follows:

- Human premolars with presence of sclerotic dentin.

**Figure 1:**
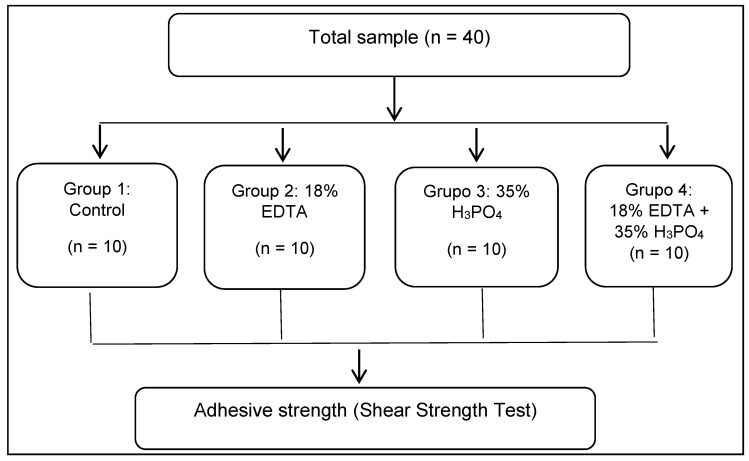
Random distribution of groups according to sample size.

The groups were formed as follows (Figure 1):

- Group 1: Control (without dentin conditioner).
- Group 2: Dentin conditioned with 18% EDTA gel (Ultradent Products, South Jordan, UT, USA).
- Group 3: Dentin conditioned with 35% H_3_PO_4_ (Ultra-Etch, Ultradent Products, South Jordan, UT, USA).
- Group 4: Dentin conditioned with 18% EDTA gel plus 35% H_3_PO_4_.

### 2.3. Sample Characteristics and Preparation

Remains of soft tissue or bacterial plaque were removed from the teeth with an ultrasonic dental scaler (DTE D5 LED, Woodpecker, Guilin, Guangxi, China). The teeth were then washed and immersed in a 1% t-chloramine solution (Milipore, Supelco, Lima, Peru) for one week for disinfection. They were then placed in a container with distilled water at 4 °C for maintenance, changing the water every 7 days. The 40 sample units were placed in saline solution for 24 h at 37 °C ± 2 °C before sectioning the occlusal third of the crown.

### 2.4. Dentin Conditioning and Resin Composite Bonding

The sample was divided into four groups, and a single operator proceeded to cut the occlusal third with a low-speed micromotor (Strong 210, Saeshin, Korea) and a low-speed water-cooled diamond cutting disc (DREMEL^®^ 300 Series, Mt. Prospect, IL, USA). After the dentin was exposed, a total-etch adhesive (Tetric^®^ N-Bond, Ivoclar Vivadent AG, Schaan, Liechtenstein) was placed and light cured for 20 s. Then, a block of Tetric^®^ N-Ceram Bulk-Fill resin composite color A2 (Ivoclar Vivadent AG, Schaan, Liechtenstein) made from a standard mold with 4 × 4 mm surface area was applied to the dentin surface and light cured for 10 s. According to the material safety data sheets, Tetric^®^ N-Ceram Bulk-Fill resin composite contains bis-GMA, bis-EMA, UDMA plus barium silicate aluminous glass, “isofiller” (prepolymer, glass, and ytterbium fluoride), ytterbium fluoride and mixed oxides. The block dimensions were measured with a WHO periodontal probe (Hu Friedy, Chicago, IL, USA), and the diamond cutting disc was changed in each sample unit.

**Figure 2:**
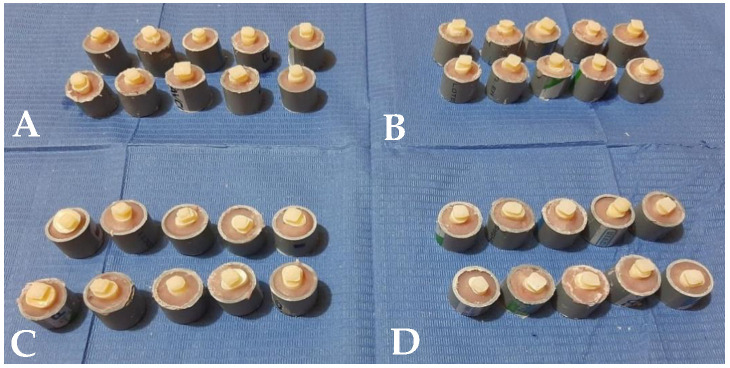
Sample units according to study group. (**A**) No dentin conditioning (Control), (**B**) dentin conditioned with 18% EDTA, (**C**) dentin conditioned with 35% H_3_PO_4_ and (**D**) dentin conditioned with 18% EDTA plus 35% H_3_PO_4_.

**Figure 3:**
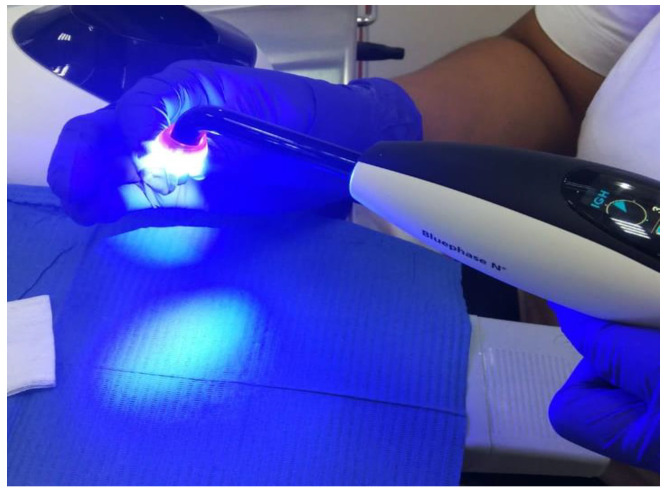
Light curing of Bulk-Fill resin blocks with LED unit.

The procedure for each group was as follows (Figure 2):

- Group 1: No dentin etching. Only rinsed in water for 5 s, and excess moisture was dried with sterile gauze. Then, a layer of Tetric^®^ N-Bond adhesive was placed with a microbrush, and air was gently applied for 5 s. Finally, a block of Tetric^®^ N-Ceram Bulk-Fill resin composite color A2 was placed and light cured perpendicularly for 10 s at a maximum distance of 1 mm to the upper surface with a Bluephase N LED (Ivoclar Vivadent AG, Schaan, Liechtenstein) at an intensity of 1200 mW/cm^2^ for 10 s. (Figure 3). The light intensity of curing unit was previously verified using a radiometer (Bluephase Meter II, Ivoclar Vivadent AG, Schaan, Liechtenstein).
- Group 2: Dentin etching was performed with 18% EDTA gel for 90 s. Then it was washed with water for 10 s, and the excess moisture was dried with sterile gauze. Then, the adhesive was applied, and the resin composite was placed using the same procedure as group 1 [5].
- Group 3: Dentin etching was performed with 35% H_3_PO_4_ gel for 15 s. Then, it was washed with water for 10 s, and the excess moisture was dried with sterile gauze. Then, the adhesive was applied, and the resin composite was placed using the same procedure as groups 1 and 2.
- Group 4: Dentin etching was performed with 18% EDTA gel for 90 s. Then, it was washed with water for 10 s, and the excess moisture was dried with sterile gauze. Then, 35% H_3_PO_4_ gel was applied for 15 s. Then, it was again washed with water, and excess moisture was dried with sterile gauze. Then, the adhesive was applied, and the resin composite was placed using the same procedure as in groups 1, 2 and 3.

Subsequently, 10,000 thermocycles between 5 +/− 2 °C and 55 +/− 2 °C were applied to all sample units. The exposure to each bath was 30 s, and the transfer time between baths was 10 s. [29]

### 2.5. Shear Strength Test

**Figure 4:**
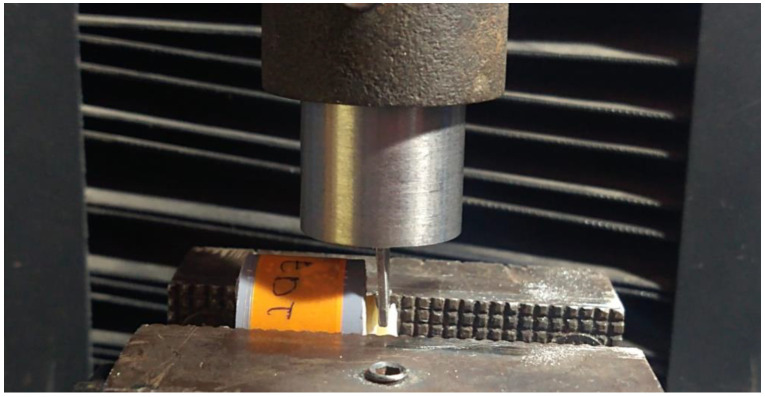
Shear test with universal testing machine.

The roots of the 40 sample units were immersed in self-curing acrylic (Vitacryl, Vitalloy, Lima, Peru) in cylindrical molds to facilitate their handling in the shear test. The 40 prepared samples were subjected to a shear strength study using a universal testing machine (CMT-5L, 7419 series, Liangong Group, Jinan, Shandong, China) with digital software (Smart Test) at a crosshead speed of 0.75 mm/min. A 1 mm wide bevel cutting bar located on the upper head of the universal testing machine was used. This head, when descending at the indicated speed, came into contact with the sample resin–dentin junction located on the lower head. As the bar descended, it exerted a force (Newton) that was counteracted by the resistance (MPa) provided by the resin–dentin bond. This force reached its maximum value when the separation between resin composite and dentin occurred. To obtain the bond strength values in megapascals (MPa), the shear stress formula was used: R = F/A, where R is strength, F is the force in newtons obtained with the universal testing machine and A is the bond area expressed in mm2 and constitutes the worked area (Figure 4).

### 2.6. Statistical Analysis

Data were entered into a Microsoft Excel 2019^®^ tab and subsequently imported into SPSS (Statistical Package for the Social Sciences Inc., IBM, Armonk, NY, USA) version 28.0 for statistical analysis. For descriptive analysis, measures of central tendency (mean) and dispersion (standard deviation) were used. For comparative analysis, Shapiro–Wilk’s statistical assumptions of normality and Levene’s homoscedasticity and randomness based on the Wald–Wolfowitz mean were previously tested. Based on these results, a statistical decision was made to use the parametric one-factor intergroup ANOVA test with Tukey’s post hoc test. All analyses were performed considering a significance level of 5% (*p* < 0.05).

### 2.7. Ethical Considerations

This research respected the bioethical principles for medical research with human beings of the Declaration of Helsinki. This research was approved by the Ethics and Research Committee of the School of Stomatology of the Universidad Privada San Juan Bautista with approval letter No. 1410-2021-CIEI-UPSJB. The teeth obtained in the present investigation were donated by the patients, with prior informed consent.

## 3. Results

The group without dentin conditioning presented the lowest average adhesive strength with 5.54 ± 0.88 MPa, while the group with dentin conditioning based on 18% EDTA plus 35% H_3_PO_4_ presented the highest average adhesive strength with 8.72 ± 1.02 MPa (Table 1).

**Figure 5:**
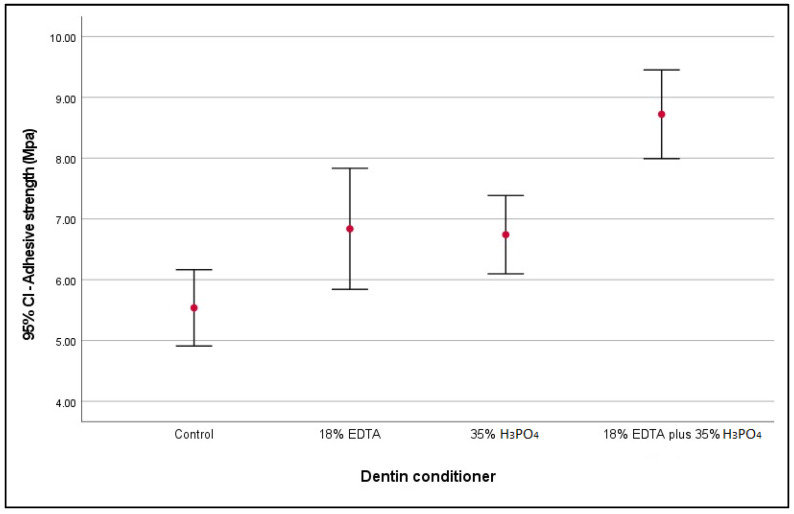
Comparison of means at 95% CI of adhesive strength (MPa) according to conditioner applied.

When comparing the adhesive strength according to the dentin conditioning applied, significant differences (*p* < 0.001) were observed between groups (Table 2). Therefore, multiple comparisons showed that 18% EDTA and 18% EDTA plus 35% H_3_PO_4_ presented significantly higher adhesive strength compared to the group that did not receive dentin conditioning (control) (*p* = 0.047 and *p* < 0.001, respectively). In addition, the group with 18% EDTA plus 35% H_3_PO_4_ conditioning showed significantly higher adhesive strength compared to groups conditioned with 18% EDTA (*p* = 0.002) and 35% H_3_PO_4_ (*p* = 0.001) (Table 3 and Figure 5).

## 4. Discussion

The acid conditioning of dentin and the application of primers as bonding agents activate metalloproteinases (MMPs) [8,9,10,15,16,17,27], which are cell-derived proteolytic enzymes responsible for the degradation of collagen fibers [30]. The degradation of collagen within the hybrid layer by MMPs is a vulnerable point for modern adhesive systems. One way to minimize and prevent the release of MMPs is to use a neutral conditioning agent, such as EDTA [3,6,7,13], that dissolves the extra- and intra-fibrillar minerals. This process exposes the collagen fibers and causes neutral dehydration of dentin. A hybrid layer is then formed by priming, allowing for better resin composite infiltration [7,13,31]. Therefore, the present study aimed to compare which of the conditioning agents commonly used, such as 18% EDTA or 35% H_3_PO_4_, offered better adhesive strength in dentin when using resin composites. According to the results, the null hypothesis was not rejected since the adhesive strength in dentin conditioned with 18% EDTA did not show significant differences when compared to 35% phosphoric acid, but it is worth mentioning that applying 18% EDTA followed by 35% H_3_PO_4_ produced a significantly higher adhesive strength compared to these conditioners used separately.

Imbery et al. [13] reported that 17% EDTA gel applied for 90 s on artificially aged dentin showed significantly higher adhesive strength values compared to 37.5% H_3_PO_4_. This was discordant with what was obtained in the present study since no significant differences in adhesive strength were found between EDTA and H_3_PO_4_ as dentin conditioning agents. This discrepancy may be due to the different concentrations used or the technique used for artificial aging. In the present study, a technique of 10,000 thermal cycles equivalent to 1 year of clinical aging was applied. In contrast, Imbery et al. [13], Kim et al. [6] and Deng et al. [32] used sodium hypochlorite (NaClO) between 10% and 12% for 1 to 3 h as a method of artificial aging, which is equivalent to 60,000 thermal cycles (6 years of aging) [29,33]. It should be noted that these differences could also be associated with other factors, such as the use of a different type of resin composite, the pH of the adhesive and the use of a higher light intensity (1200 mW/cm^2^), for curing the adhesive and resin composite system.

Kim et al. [6] and Imbery et al., [13] explained from a biological approach that the significant values of adhesive strength obtained when dentin is treated with EDTA may be due to its almost neutral pH, which would help prevent the release of MMPs. When the pH of conditioning agents is lower than 4.5, as in H_3_PO_4_ (pH = 0.6), and these agents come into contact with MMPs, chemical reactions occur that engage calcium ions, zinc and extracellular proteins of the glycoprotein family that pair with different MMPs to block their catalytic damage [13,31]. In addition, the four carboxylic groups of EDTA sequester metal ions from dentin and cause selective or partial dissolution of hydroxyapatite [1,13], leaving residual apatite crystals in the collagen matrix and making it more resistant to denaturation [1,34,35]. Finally, EDTA could favor the stability of collagen fibers by removing the surface smear layer and allowing for the penetration of acidic primers creating a cleaner substrate with a more retentive etching pattern. [1,13,26,36]

In vitro and in vivo studies [6,14,37,38,39] explained that the decrease in resin–dentin adhesive strength and collagen degradation occur with the passage of time or by other artificial aging treatments. Because of this, it could be presumed that the 10,000 thermal cycles applied in the present study were not sufficient to compare the adhesive strength of EDTA versus H_3_PO_4_ on dentin since no significant differences were found between them. This is in agreement with the results of Kim et al. [6], who reported that the immediate adhesive strength values of the EDTA-treated group did not differ significantly from those of the H_3_PO_4_-treated group. However, it is likely that not only the artificial aging factor could be related to the similar adhesive strength values between EDTA and H_3_PO_4_, but also the composition of the adhesive since the Tetric N Bond used in our study does not contain polyalkenoic acid [40,41], which has been reported to improve adhesive strength when dentin is preconditioned with H_3_PO_4_. This is based on the fact that the carboxylic group of polyalkenoic acid and dentin hydroxyapatite incompletely dissolved by H_3_PO_4_ could form an ionic bond with high adhesive strength between the resin composite and dentin [6,42].

It is worth mentioning that Jaques et al. [7] reported that the use of 0.5 M or 18% EDTA (pH = 7.2) with subsequent application of Clearfil SE Bond self-etching adhesive (pH = 2), as well as the application of EDTA with prior conditioning using acidic primer and conventional single bond adhesive (pH = 4.3), showed very high adhesive strength values. Therefore, it can be deduced that EDTA, being a mild etchant, requires the help of a more acidic agent, such as an acidic primer or self-etching adhesives [7,24] with very high pH (pH: 2–3), to sufficiently demineralize the dentin, unlike full conditioning adhesives that generally have pH >5 [7,8,9].

To date (September 2022), no literature has been found that assesses adhesive strength when using 18% EDTA as dentin conditioner with complementary application of 35% H_3_PO_4_. The importance of the present study lies in the novel finding of applying these two conditioning agents in bulk-fill resin composites in a complementary manner and obtaining dentin adhesive strength values significantly higher than the results of the same conditioners used separately. This is probably because conditioning the dentin first with EDTA would cause inhibition of MMPs, allowing for the partial removal of the smear layer in the dentin tubules without causing damage to the dentin collagen fibers. In addition, EDTA is likely to neutralize the very low pH of the H_3_PO_4_ subsequently applied as a conditioning protocol, thus attenuating the formation of MMPs for a measured exposure of the collagen fibers with wide intrafibrillar spaces [7,13,39] and favoring a micromechanical adhesion [43,44] of the resin composite. Other authors [8,27] have agreed with the results obtained in the present study, pointing out the importance of new bonding systems providing long-lasting MMP inhibitory capabilities to preserve the integrity of the hybrid layer and improve the durability of the dentin–resin composite bond. Therefore, it is advisable to continue this line of research with scanning electron microscopy, Fourier transform infrared spectrometry (FTIR) or X-ray photoemission spectroscopy (XPS) studies to assess microstructural changes or dentin topography after etching in order to complement and reinforce the results obtained for adhesive strength in bulk-fill resin composites when dentin was conditioned with EDTA followed by H_3_PO_4_.

The methodology used in the present study is based on scientific precedents in terms of sample size, clear protocols for sample preparation, use of materials according to precedents and calibrated instruments to assess adhesive strength, among others. All this allowed us to reduce biases and strengthen the design. However, it should be recognized within the limitations of the present study that the data obtained should be taken with caution since this was an in vitro study and cannot be extrapolated to the clinical field. Despite the aforementioned limitations, this lays the groundwork for recommending future in vitro or in vivo studies with prolonged follow-up that focus on assessing dentin adhesive strength by applying EDTA 18% and then complementing it with 35% H_3_PO_4_ at different times under different artificial aging techniques, such as thermal cycling [7,31,32] or sample immersion in 10–13% sodium hypochlorite solution for 1 to 3 h [6,13,31,32] and using self-etch and total conditioning adhesive systems [7,45].

## 5. Conclusions

In summary, with the limitations of the present in vitro study, it can be concluded that the adhesion of the bulk-fill resin composite to dentin was favorable when preconditioning was performed using 18% EDTA followed by 35% H_3_PO_4_. In contrast, when both etchants were used separately, the bulk-fill resin composite showed similar bond strength values in both cases, but significantly lower compared to their sequential application.

## Figures and Tables

**Table 1:** Descriptive values of adhesive strength (MPa) according to type of conditioner used.

Conditioner	n	Mean	SD	SE	95% CI		Min	Max
					LL	UL		
**Control**	10	5.54	0.88	0.28	4.91	6.17	4.04	6.75
**18% EDTA**	10	6.84	1.39	0.44	5.84	7.83	4.95	9.41
**35% H_3_PO_4_**	10	6.74	0.90	0.28	6.10	7.39	5.24	7.80
**18% EDTA plus 35% H_3_PO_4_**	10	8.72	1.02	0.32	7.99	9.45	7.52	10.51

n: sample size; SD: standard deviation; SE: standard error of mean; 95% CI: 95% confidence interval; LL: lower limit, UL: upper limit; Min: minimum value; Max: maximum value.

**Table 2:** Comparison of adhesive strength (MPa) according to type of conditioner used.

Conditioner	n	Mean	SE	95% CI		* *p*	** *p*	*** *p*
				LL	UL			
**Control**	10	5.54	0.28	4.91	6.17	0.756	0.333	<0.001
**18% EDTA**	10	6.84	0.44	5.84	7.83	0.907		
**35% H_3_PO_4_**	10	6.74	0.28	6.10	7.39	0.480		
**18% EDTA plus 35% H_3_PO_4_**	10	8.72	0.32	7.99	9.45	0.440		

n: sample size; SE: Standard error of mean; 95% CI: 95% confidence interval, UL: upper limit, LL: lower limit; * Based on Shapiro–Wilk test: normal distribution (*p* > 0.05); ** Based on Levene’s test: homogeneous variances (*p* > 0.05); *** Intergroup one-factor ANOVA test: significant differences (*p* < 0.05).

**Table 3:** Multiple comparison of adhesive strength (MPa) according to type of conditioner used.

Conditioner		MD	SE	95% CI		*p* *
				LL	UL	
**Control**	18% EDTA	−1.3	0.48	−2.59	−0.01	0.047
	35% H_3_PO_4_	−1.2	0.48	−2.49	0.08	0.073
	18% EDTA plus 35% H_3_PO_4_	−3.18	0.48	−4.47	−1.90	<0.001
**18% EDTA**	35% H_3_PO_4_	0.09	0.48	−1.19	1.38	0.997
	18% EDTA plus 35% H_3_PO_4_	−1.88	0.48	−3.17	−0.60	0.002
**35% H_3_PO_4_**	18% EDTA plus 35% H_3_PO_4_	−1.98	0.48	−3.27	−0.69	0.001

MD: mean difference; SE: standard error; 95% CI: 95% confidence interval, UL: upper limit, LL: lower limit; * Based on Tukey’s post hoc: *p* < 0.05 (significant differences).

## Data Availability

The data presented in this study are available on request from the corresponding author.
